# Effects of thinning on the structure of soil microbial communities in a subtropical secondary evergreen broad-leaved forest

**DOI:** 10.3389/fpls.2024.1465237

**Published:** 2024-11-25

**Authors:** Liangjin Yao, Jiejie Jiao, Chuping Wu, Bo Jiang, Lili Fan

**Affiliations:** ^1^ Zhejiang Academy of Forestry, Forest Ecology Innovation Team, Hangzhou, China; ^2^ Research Institute of Subtropical Forestry, Chinese Academy of Forestry, Hangzhou, China

**Keywords:** thinning, rhizosphere soil, 16S rDNA, soil microbial communities, soil nutrient cycling

## Abstract

**Introduction:**

Thinning is a common practice to enhance tree growth, but its effect on rhizosphere soil microorganisms in subtropical secondary evergreen broadleaved forests remains unclear.

**Methods:**

This study used 16S rDNA amplicon sequencing to explore soil microflora of five shrubs and five tree species.

**Results:**

The results showed that thinning altered nutrient distribution and pH in rhizosphere soil, impacting microbial richness, which varied by tree species. The dominant bacterial phyla were Acidobacteria, Proteobacteria, Actinobacteria, and Firmicutes. Although the dominant microbial species remained largely unchanged, thinning increased the relative abundance of Firmicutes. Thinning intensity between 10-15% significantly altered the structure of soil microbial communities, demonstrating species-specific responses.

**Discussion:**

These changes in microbial structure may influence tree growth. This study proposed the potential effects of thinning on rhizosphere soil microorganisms and suggests future research to investigate the specific microbial mechanisms affected by thinning.

## 1 Introduction

Thinning is a crucial forest management strategy used to enhance forest biodiversity, improve wood quality, and maintain ecosystem function (Davies et al., 2023; Lee et al., 2024). It can regulate the stand environment, nutrient cycling, forest productivity, and tree quality. Most traditional research has focused on soil nutrients and plant community structure (Zhou et al., 2019; Camponi et al., 2022), while microbial communities involved in nutrient cycling remain underexplored (Zhang et al., 2023). This gap limits our understanding of forest management affects forest ecological functions.

Soil microbiota performs key ecosystem functions, including biogeochemical processes such as litter decomposition, by converting soil materials into nutrients (Wang et al., 2021; Liu et al., 2024). Root microbial communities affect tree health and soil nutrient cycling, varying significantly between tree species due to physiological traits and root exudates. For example, poplar and pine have distinct rhizosphere microbial profiles, with poplar dominated by *Actinobacteria* and pine dominated by mycorrhizal fungi. These differences highlight the importance of understanding species-specific microbial interactions. Some studies have suggested that thinning can affect the rhizosphere microbial structure (Qiang et al., 2023; Zhang et al., 2023). Thinning influences the composition and function of root microorganisms by changing environmental factors such as soil pH, nutrient content and moisture. However, the complexity of tree environments and species diversity suggests that the effects of thinning on rhizosphere soil microflora remain unclear.

Middle subtropical evergreen broad-leaved forest, a typical forest vegetation types in China, is characterized by complex species composition and significant ecosystem service functions, playing a key role in ecosystem security and stability (Edwards et al., 2014; Cortés-Calderón et al., 2021). However, these forests have been degraded by long-term anthropogenic disturbances, such as logging and land-use changes, resulting in compromised ecological functions, particularly in secondary forests of Zhejiang Province (Shang et al., 2014). This study was conducted in the Wuchaoshan National Forest Park in Hangzhou between October 2020 and June 2021 according to the Center for Tropical Forest Science (CTFS) standard to establish a 6-hectare dynamic monitoring prototype.

In this study, we investigated the effects of 10-15% thinning on the microbial communities of tree rhizosphere soil by analyzing five shrubs and five tree species in Wuchao Mountain using *16S rDNA* sequencing. Our objective was to systematically analyze the differences in the composition and structure of rhizosphere microbial communities in different tree species and to explore the underlying mechanisms driving those differences. We assessed how environmental factors such as soil pH, nutrient content, humidity, and temperature, regulate these microbial communities and investigated their adaptive and dynamic responses to changing environmental conditions. We hypothesized that microorganisms may mediate the effects of thinning on tree growth. This study underscores the importance of considering microbial dynamics in forest management practices. Future research should focus on quantifying these microbial changes and their specific impact on tree growth to inform targeted forest management and restoration strategies.

## 2 Materials and methods

### 2.1 Profile of study area

**Figure 1:**
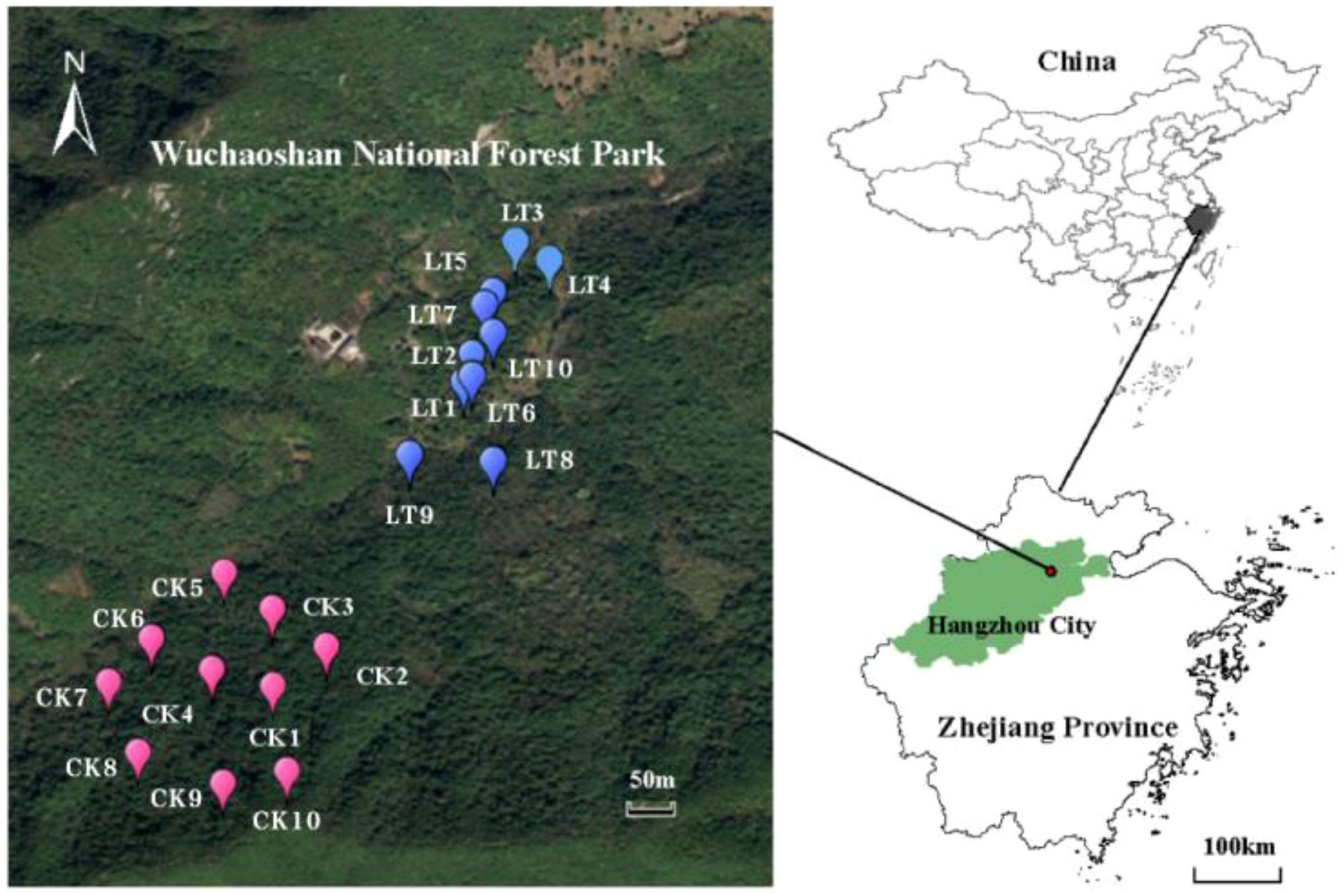
locations of the study sites in Hangzhou city, Zhejiang province, China. LT represents plots with 10%-15% thinning, while CK represents control plots with dominant trees and shrubs, without thinning.

Wuchao Mountain National Forest Park belongs to the remnant of Wuchao Mountain Range. It is located in Xianlin Town (33°41’N, 120°00’E), Yuhang District, Hangzhou City, Zhejiang Province, about 20 km away from Hangzhou city (**Figure 1**). The park covers an area of 522 hectares, with an average altitude of 264 m and the maximum altitude of 494.7 m. It is a natural forest ecosystem situated on the city’s outskirts. Positioned in the middle subtropical zone, the park benefits from favorable natural conditions such as water and heat. The average annual temperature is 16.1°C, the average temperature of the coldest month January is 3.6°C, and the average temperature of the hottest month August is 38.4°C. The annual rainfall was 1 400.7 mm, with evaporation in July and August exceeding the rainfall by 79.6 mm. The annual sunshine duration reached 1 970.6 hours, and the plant growth period lasted 311 days. The predominant soil types in the park are red soil and yellow soil, both of which are typical for this region and contribute to the area’s rich nutrient profile. Wuchao Mountain National Forest Park boasts a forest coverage rate of 93% and a rich diversity of plant species.

### 2.2 Sample site setting and investigation

In this study, according to the principles set by *Center for Tropical Forest Science* (CTFS) standard, we established 20 plots (20 m × 20 m each) within a forest area characterized by similar elevation, slope, and other habitat characteristics in 2020. These plots were chosen to ensure consistency in environmental conditions across the study area. Within each plot, we recorded the diameter of all woody tree species with a diameter at breast height (DBH) of≥1 cm, along with measurements of tree height and spatial coordinates. In August 2020, ten of these plots were subjected to various experimental treatments, while the remaining 10 plots served as controls.

The study was conducted in the Wuchao Mountain Forest, a region that is currently in the mid to late stages of ecological succession. This area is marked by a diverse plant community, with the top five species by importance value being *Schima superba*, *Camellia fraterna*, *Symplocos anomala*, *Cyclobalanopsis glauca*, and *Eurya rubiginosa*, listed in in descending order of importance. *Schima superba* tends to cluster at higher elevations within the plots, with larger diameter individuals primarily distributed on the middle to upper slopes. In contrast, *Camellia fraterna* and *Symplocos anomala* are predominantly found in relatively flat, low-elevation areas, while *Eurya rubiginosa* is more common on higher elevation slopes. *Cyclobalanopsis glauca* and *Cunninghamia lanceolata* are generally found on low-elevation gentle slopes where *Schima superba* is less abundant.

The soil in Wuchao Mountain is predominantly red soil, characterized by a relatively high humus layer and rich nutrient content, supporting the diverse vegetation. The forest’s soil profile and nutrient composition are indicative of the region’s late-successional stage, contributing to the observed species distribution patterns A comprehensive plant community assessment was conducted for all plots in August 2022. The assessment was scheduled following several consecutive days of clear weather to ensure that soil conditions were stable and not influenced by recent rainfall, allowing for more accurate measurements and observations.

### 2.3 Thinning and sampling method

At the beginning of the experiment, we collected and measured 45 species of main woody plants in the secondary evergreen broad-leaved forest. The main species in the community were divided into four functional groups according to the forest succession process: early pioneer species, middle neutral species, middle and late species and late shade-tolerant species. In August 2020, considering the ecological characteristics and wood value of the tree species, they were further classified into three groups for thinning experiments: target species, auxiliary species, and clearing species (**Table 1**).

**Table 1:** Classification of target species, auxiliary species, and cleared species.

Categories	Species	Feature description
Purpose species of trees	*Schima superba*、*Cyclobalanopsis glauca*、*Sassafras tzumu*、*Ilex chinensis*、*Castanopsis sclerophylla* et al.	An ecological key species in the middle of the succession Tree species with high economic value, ornamental value and ecological value
Accessory species	*Camellia fraterna*、*Symplocos anomala*、*Osmanthus cooperi*、*Ilex rotunda* et al.	A tree species that is positively connected to the target tree species Tree species that can help restore forest windows
Clear tree species	*Photinia parvifolia*、*Symplocos paniculata*、*Premna microphylla*、*Rhododendron ovatum* et al.	A tree species that is negatively associated with the target tree species Tree species with great interference with the growth of the target tree species

Target species were retained, clearing tree species were completely removed, and the treatment of auxiliary tree species was determined based on the growth status and potential interference with target tree species. Ten plots were thinned at 10% -15% intensity (LT), while the other ten plots without thinning were left unthinned as controls (CK) (**Table 2**). Thinning intensity was calculated by measuring changes in diameter at breast height (DBH) per unit area before and after treatment. To avoid excessive thinning, only individuals with a DBH of 20 cm or more were thinned, with all larger trees retained. The observed decrease in the importance values of some target species may be attributed to the removal of initiation strips.

**Table 2:** Overview of sample plots with different thinning intensities of tending in secondary evergreen broad-leaved forests.

Groups	Thinning intensity (%)	Number of plants (per ha)			Basal area (m^2^/ha)			Average annual production rate (%)
		2020		2022	2020		2022	
		Pre-tending	Post-tending		Pre-tending	Post-tending		
CK	0	6465	–	5880	36.56	–	37.90	1.84
TK1	10-15	6738	3896	3978	37.07	32.03	35.50	5.42

### 2.4 Sample collection

In June 2022, root soil samples were collected from both the 10%-15% thinning plots and the unthinned control plots. For each treatment group, five trees and shrubs were selected, and multiple root tip samples were collected from five species of trees and shrubs, with at least five root tips per sample to ensure representativeness and statistical validity. Rhizosphere soil was sampled from soil approximately 1-2 millimeters around the root surface to capture soil closely associated with the microbial activity. Care was taken to avoid contamination from non-rhizosphere soil, and soil moisture at the time of collection was maintained to reflect natural conditions.

In the secondary evergreen broad-leaved forest plots, ten well-established adult trees with a DBH of about 15 cm were randomly selected from each plot. Rhizosphere soil from ten trees in the same plot was combined, mixed thoroughly, and then pooled with soil from three other plots. The composite soil was divided into four replicates and transported to the laboratory at 4°C for physical and chemical property analysis and DNA extraction.

### 2.5 Analysis of soil physical and chemical properties

Soil moisture content was determined using the oven-drying method. Minimum water holding capacity was measured using the field capacity method, while maximum water holding capacity was assessed using the complete saturation method. Soil bulk density was measured using the core sampling method, and pH was measured by the potentiometric method. Soil organic matter was determined via potassium dichromate oxidation-external heating method. Total phosphorus was measured using the acid digestion-molybdenum blue colorimetric method, total nitrogen by the Kjeldahl method, available potassium by ammonium acetate extraction, available phosphorus by olsen method, and alkali-hydrolyzed nitrogen by the alkalolytic diffusion method.

### 2.6 DNA extraction, *16S rRNA* gene amplification and MiSeq sequencing

For DNA extraction, 0.25 g of mixed soil sample were processed using the FAST DNA Kit following the manufacturer’s instructions. The integrity of extracted DNA was verified by agarose gel electrophoresis, and the DNA was immediately used for subsequent experiments.

DNA quantification was performed using the Qubit 3.0 DNA Assay Kit prior to polymerase chain reaction (PCR) amplification. The V3-V4 region of the 16S rRNA gene was amplified using the universal primers 341F (5’-CCTACGGGNGGCWGCAG-3’) and 805R (5’-GACTACHVGGGTATCTAATCC-3’), with barcodes and adapters integrated between the adapter and forward primers.

PCR amplification was conducted in two stages. The first stage involved a 30 µL reaction system containing 15 µL of 2x Taq Master Mix (Vazyme), 1 µL of 10 µM Bar-PCR forward primer, 1 µL of 10 µM reverse primer, 10–20 ng of genomic DNA, and sterile double-distilled water. The PCR conditions were: initial denaturation at 94°C for 3 minutes, followed by 5 cycles of denaturation at 94°C for 30 seconds, annealing at 45°C for 20 seconds, and extension at 65°C for 30 seconds, and then 20 cycles of denaturation at 94°C for 20 seconds, annealing at 55°C for 20 seconds, and extension at 72°C for 30 seconds, with a final extension at 72°C for 10 minutes.

The second stage of PCR amplification, adding Illumina bridge PCR-compatible primers, used similar conditions with adjustments: initial denaturation at 95°C for 3 minutes, followed by 5 cycles of denaturation at 94°C for 20 seconds, annealing at 55°C for 20 seconds, extension at 72°C for 30 seconds, and a final extension at 72°C for 5 minutes.

PCR products were visualized using 2% agarose gel electrophoresis, purified with Agencourt AMPure XP, and quantified with the Qubit 3.0 DNA Detection Kit. Equimolar amounts of DNA were then pooled and sequenced on the MiSeq platform.

### 2.7 Bioinformatics analysis

Raw reads from Illumina MiSeq sequencing were processed using CASAVA for base calling and translated into sequenced reads. Quality control of raw data was performed with Prinseq (version 0.20.4) to obtain high-quality sequences. Sequences were clustered into operational taxonomic units (OTUs) at a 97% similarity threshold using Usearch (version 5.2.236).

Microbial richness and diversity were analyzed using the Chao1 and Shannon indices, calculated with Mothur (version 1.30.1). Rarefaction curves were generated using Mothur and R, based on OTUs at 97% similarity. Taxonomic annotation of OTUs was performed using the RDP 16S Classifier at an 80% confidence threshold. The community structure was analyzed statistically at various microbial classification levels.

### 2.8 Statistical analysis

Paired t-tests were used to compare rhizosphere soil microbial diversity, richness, and bacterial abundance before and after thinning. Statistical significance was set at P<0.05. All analyses were conducted using SPSS software (version 20.0; SPSS Inc., Chicago, IL). Data visualization was performed using R (version 3.6.3; R Foundation for Statistical Computing) and Prism software (version 8.0; Graph Prism Software Inc., CA, USA).

## 3 Results

### 3.1 Changes in soil physicochemical properties following thinning in a subtropical secondary evergreen broad-leaved forest

**Figure 2:**
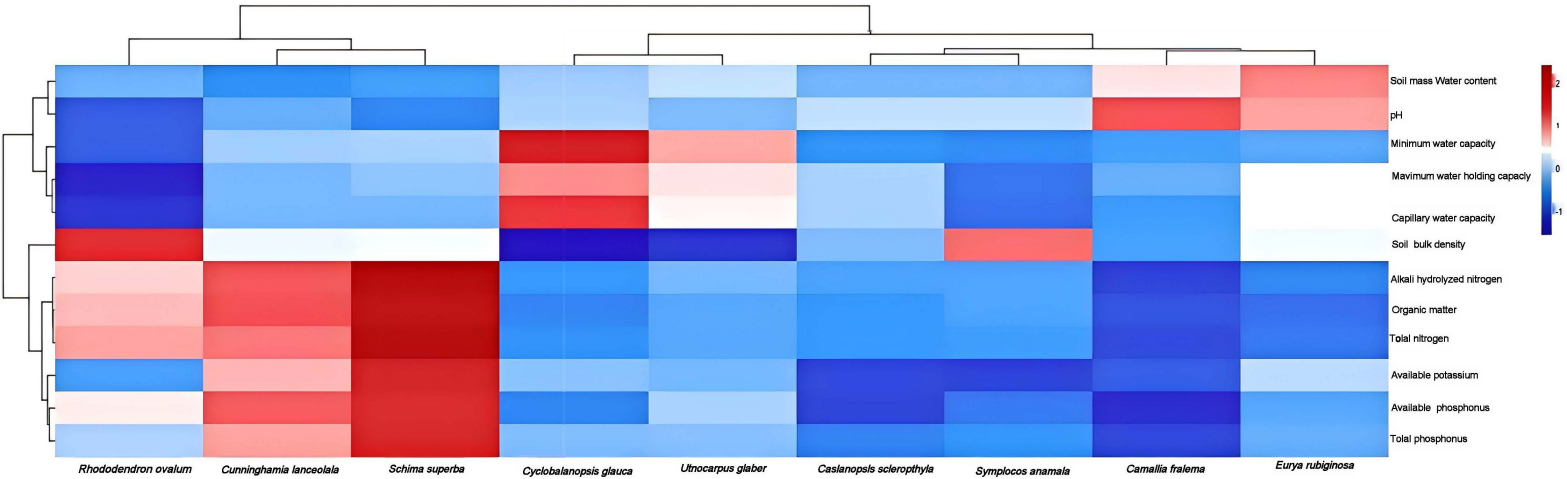
Analysis of basic physicochemical properties of rhizosphere soil of different tree species. The x-axis displays tree species, while the y-axis lists physicochemical indicators. Color intensity reflects the values of these indicators, with deeper red indicating higher values and lighter blue representing lower values.

In this study, 9 types of evergreen broad-leaved forests were selected as research targets, and the basic physicochemical properties of their root soils were analyzed. The results showed that the total nitrogen content, organic matter, alkali hydrolyzed nitrogen, and available potassium of *Schima-superba* (MH) and *Cunninghamia-lanceolata* (SM) were higher than those of other species. In addition, the pH values in the rhizosphere soils of *Camellia-fraterna* (MRLRC) and *Eurya-rubiginosa* (ZJHHL) are abnormally elevated. The physical and chemical properties of rhizosphere soil varied among different shrubs. The physicochemical properties of all soil samples are shown in **Figure 2**.

### 3.2 High-throughput sequencing analysis of soil bacteria thinning in a subtropical secondary evergreen broad-leaved forest

**Figure 3:**
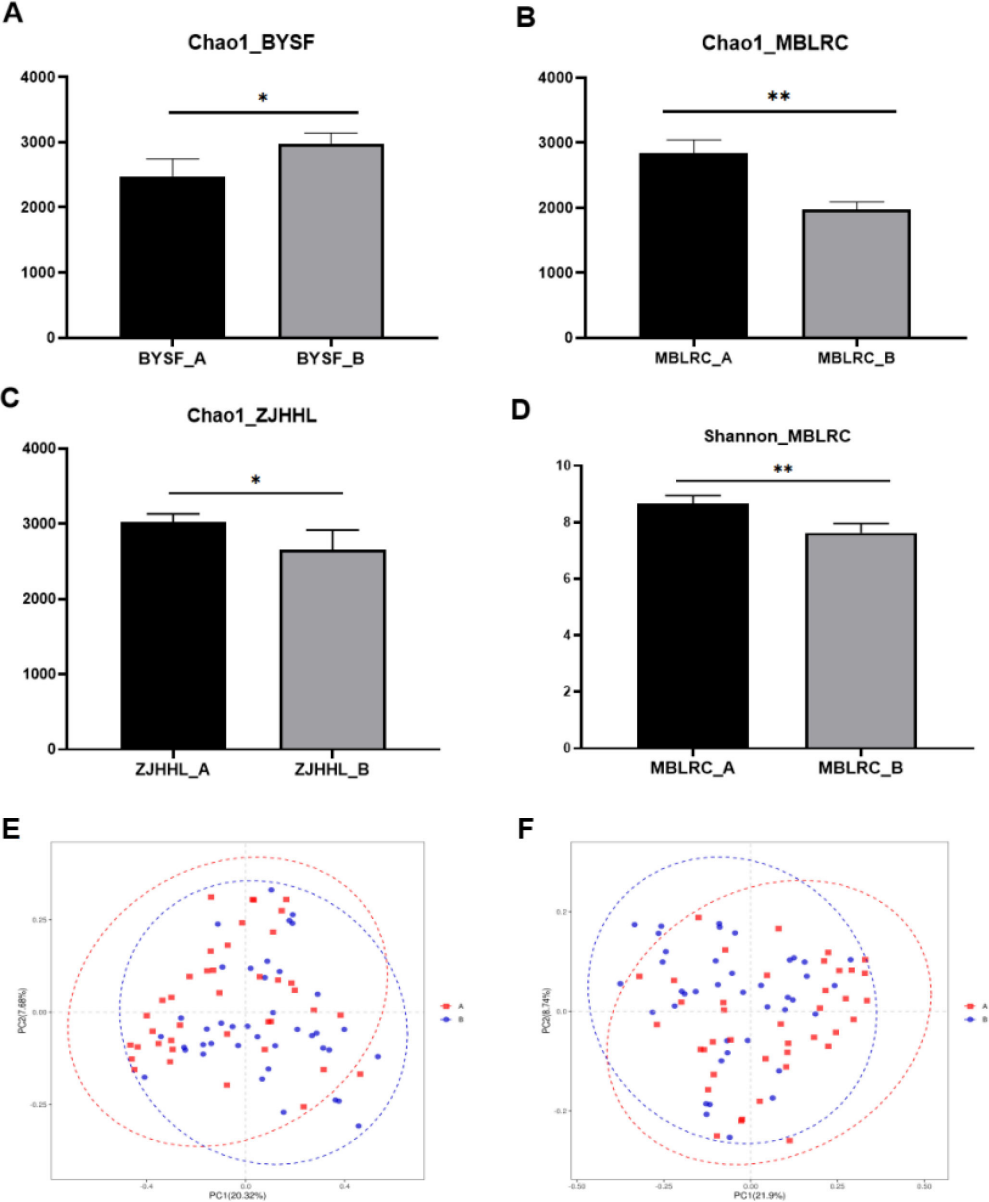
Analysis of α and β diversity of rhizosphere soil microorganisms of trees between before thinning and after thinning. **(A-D)** present the statistical analysis of changes in Chao1 and Shannon indices before and after thinning, with *P<0.05 and ** P<0.01 indicating statistically significant differences.. **(E, F)** depict the PCA and PCoA results of rhizosphere soil microorganisms, comparing conditions before and after thinning. “A” denotes conditions before thinning, while “B” represents conditions after thinning.

A total of 4537431 high quality microbial sequences were produced by Illumina Miseq with an average of 56718 sequences per sample. After OTUs clustering, we finally get 133083 OTUs, with an average of 1664 OTUs per sample. Alpha diversity analysis showed that the microbial richness and diversity varied among samples. Chao1 and Shannon indices were used to evaluate the richness and diversity of soil microbial communities across different tree species. Results showed that significant variation in rhizosphere soil microbial richness within Group A varied greatly. with rhizosphere soil microbial richness of SM exhibiting the lowest and ZJHHL the highest. In Group B, the richness of MBLRC was lowest, while *Symplocos anomala* (BYSF) was the highest. The results of α-diversity analysis of all soil samples are shown in **Figures 3A-D**.

Additionally, differences in microbial abundance were compared under different planting methods. After 10-15% thinning treatment, microbial richness of BYSF increased, while the microbial richness of MBLRC and ZJHHL decreased. For microbial diversity, ZJHHL exhibited the highest diversity and SM the lowest in Group A. In Group B, BYSF showed the highest diversity, while MBLRC had the lowest. Notably, thinning significantly reduced microbial diversity only in MBLRC, with other species showing minimal change. PCA and PCoA analysis suggested that thinning had a minimal impact on the microbial community structure in the rhizosphere soils. The results of β-diversity analysis of all soil samples are shown in **Figures 3E, F**.

### 3.3 Microbial composition analysis following thinning in a subtropical secondary evergreen broad-leaved forest

**Figure 4:**
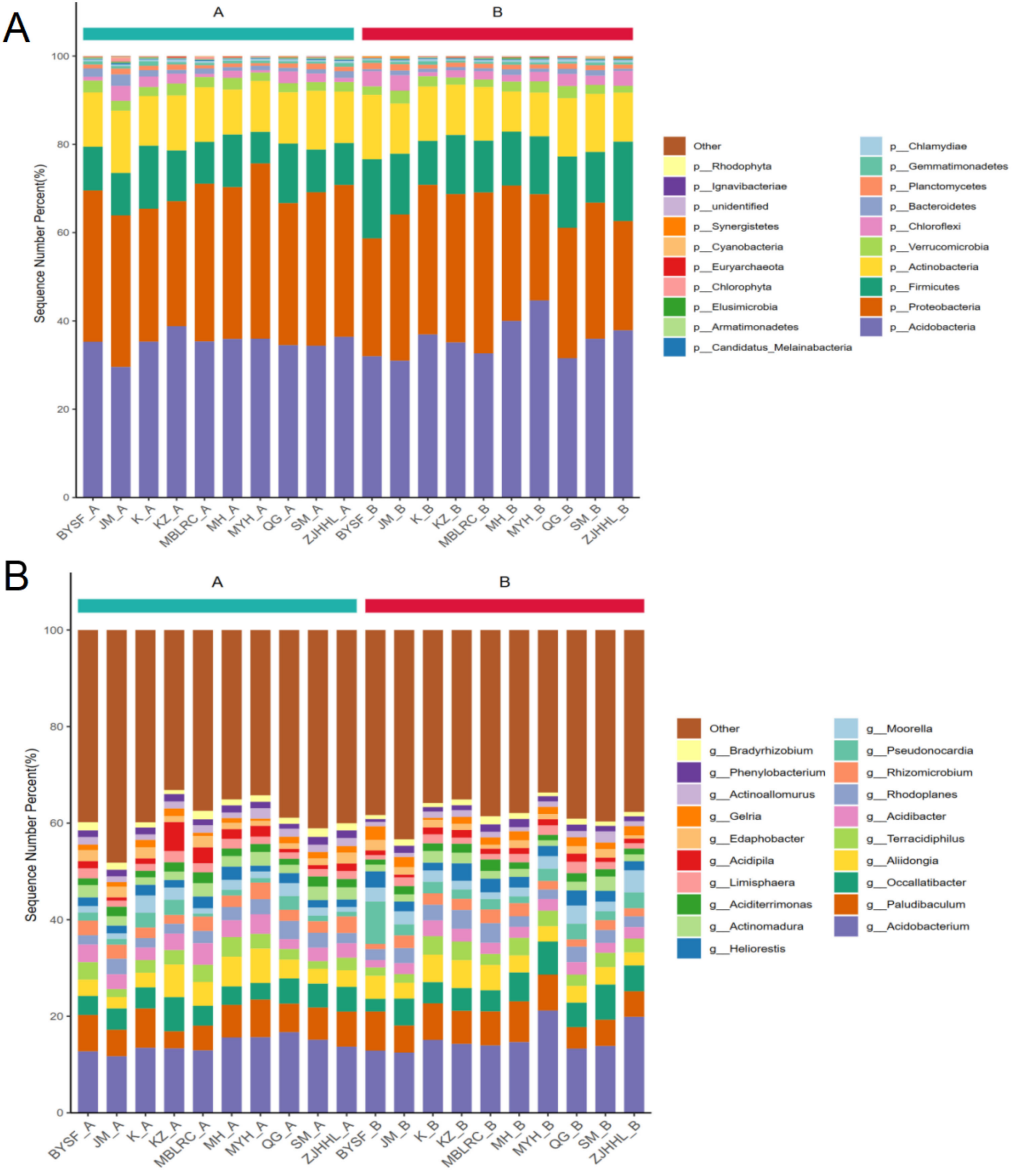
Composition of rhizosphere soil microbial communities of different tree species. **(A)** illustrates the rhizosphere soil microbial community composition at the phylum level, while **(B)** shows the composition at the genus level for various tree species. The abbreviations correspond to the following species: MH, Schima superba; SM, Cunninghamia lanceolata; MBLRC, Cunninghamia lanceolata; ZJHHL, Eurya rubiginosa; BYSF, Symplocos anomala; JM, Loropetalum chinense; KZ, Rhododendron ovatum; MYH, Castanopsis sclerophylla; K, Lithocarpus glaber; and QG, Cyclobalanopsis glauca.

We analyzed the microbial community composition of root-associated soil in different tree species under normal conditions and 10-15% thinning. The structure of rhizosphere soil microbial flora before and after thinning is shown in **Figure 4**. At the phylum level, under normal conditions, the rhizosphere soil of MBLRC, MH, SM, *Loropetalum chinense* (JM), *Rhododendron ovatum* (KZ), and ZJHHL had the highest relative abundance of Acidobacteria, with values of 35.28%, 35.30%, 38.81%, 35.92%, 34.51%, and 36.41%, respectively. The second abundant bacteria were Proteobacteria, with relative abundances of 34.27%, 30.12%, 28.29%, 34.38%, 32.18%, and 34.63%, respectively. In contrast, the rhizosphere soil of *Castanopsis sclerophylla* (MYH), BYSF, *Cyclobalanopsis glauca* (QG), and *Lithocarpus glaber* (K) had Proteobacteria as the most dominant phylum, with relative abundances of 34.33%, 35.74%, 39.69%, and 34.76%, respectively, while Acidobacteria were the second most abundant bacteria. relative abundances of 29.58%, 35.35%, 35.99%, and 34.38%, respectively. Additionally, Actinobacteria and Firmicutes also had relatively high abundances in the rhizosphere soils under normal conditions. Overall, the predominant bacteria phyla were Acidobacteria, Proteobacteria, Actinobacteria, and Firmicutes.

Under 10-15% thinning, Acidobacteria remained the most abundant phylum in the rhizosphere soil of MBLRC, MH, SM, JM, KZ and ZJHHL, with relative abundances between 31.56% and 44.64%. Followed by Proteobacteria, the relative abundance ranging from 26.69% to 33.89%. In the rhizosphere soil of MYH and BYSF, Proteobacteria had the highest the relative abundance (33.12% and 36.44%, respectively), followed by Acidobacteria (30.99% and 32.66%, respectively). In addition, *Firmicutes* and *Proteobacteria* also accounted for significant proportions of microbial communities in the rhizosphere soils under 10-15% thinning. These findings suggest that while thinning can affect the abundance of certain microbial taxa, the overall microbial composition remains relatively stable.

At the genus level, under normal conditions, *Acidobacterium* was the most prevalent genus across all tree species, with a relative abundance exceeding 10%. Paludibaculm was the second most common genus, although Occallatibacter had a higher relative abundance in the rhizosphere soils of SM compared to of Paludibaculm. In general, Acidobacterium, Paludibaculm and Occallatibacter were the dominant genera under normal conditions. Under 10-15% thinning, the main bacterial genera remained consistent, with Acidobacterium, Paludibaculm and Occallatibacter still being predominant. However, in the rhizosphere soil of KZ and K, Occallatibacter surpassed Paludibaculm, and the difference in the relative abundance, while in other samples, the order of dominance was Acidobacterium>Paludibaculm>Occallatibacter.

### 3.4 Microbial difference analysis following thinning in a subtropical secondary evergreen broad-leaved forest

**Figure 5:**
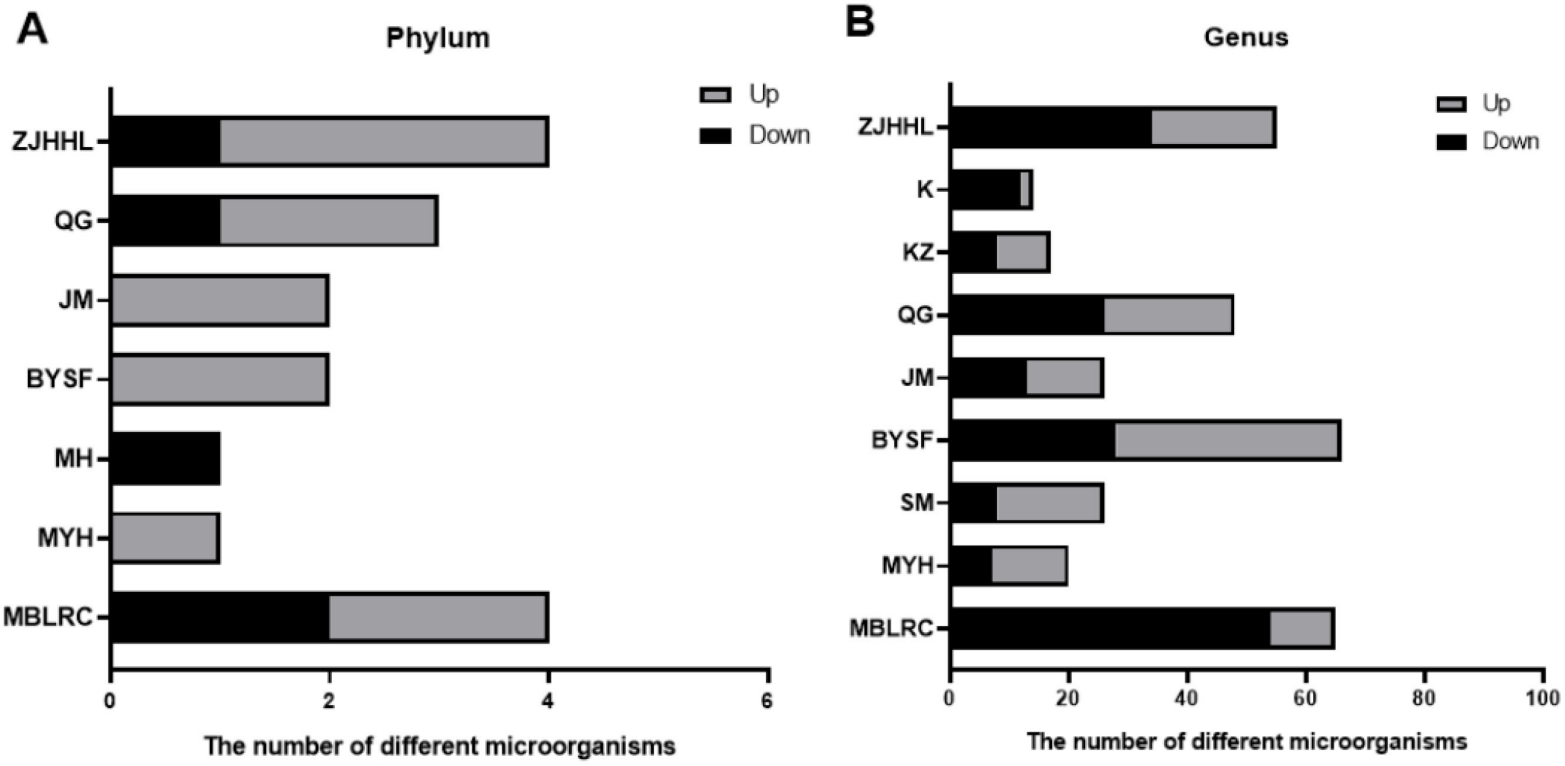
Effects of thinning on the relative abundance of rhizosphere soil microorganisms. **(A)** shows the number of bacterial species with increased or decreased relative abundance at the phylum level post-thinning, while **(B)** presents these changes at the genus level. The abbreviations correspond to the following species: MH, Schima superba; SM, Cunninghamia lanceolata; MBLRC, Cunninghamia lanceolata; ZJHHL, Eurya rubiginosa; BYSF, Symplocos anomala; JM, Loropetalum chinense; KZ, Rhododendron ovatum; MYH, Castanopsis sclerophylla; K, Lithocarpus glaber; and QG, Cyclobalanopsis glauca.

Based on *16S rDNA* amplicon sequencing, we analyzed the differences in microbial communities in the rhizosphere soil of various tree species mediated by 10-15% thinning. The difference analysis of rhizosphere soil microbial communities before and after thinning is shown in **Figure 5**. At the phylum level, significant changes were observed in. Specifically, the relative abundances of Firmicutes, Bacteroidetes, Ignavibacteriae and Thaumarchaeota changed in MBLRC; Euryarchaeota in MYH; Chloroflexi in MH; Euryarchaeota and Chloroflexi in BYSF; Elusimicrobia in JM; Firmicutes, Elusimicrobia, and Proteobacteria in QG; Firmicutes, Cyanobacteria, Chloroflexi, and Ignavibacteriae in ZJHHL. The effects of 10-15% thinning were species-specific and mainly observed in shrubs. Additionally, the bacteria primarily affected by thinning were *Firmicutes*, generally increasing in relative abundance after thinning.

At the genus level, thinning at 10-15% significantly impacted the rhizosphere soil microorganisms, with species-specific variations. Thinning resulted in differences in bacterial abundance ranging from 9 species (K) to 66 species (BYSF). For MBLRC, 11 microbial species increased and 54 species decreased, with Thermosporothrix increased and Asticcacaulis decreased. In MYH, 20 microbial species increased and 13 species decreased, with Hazenella increasing by 6.3 times and Granulibacter decreasing by 4.4 times. In SM, 26 types of microbial species varied, with Polymorphobacter increasing 27 times and Estrella decreasing eightfold. BYSF showed changes in 66 microbial species, with Thermobaculum increasing 17 times and that of Paucimonas decreasing sixfold. In JM, 26 microbial species varied, with Thermobaculum increasing 25 times and Roseomonas decreasing sevenfold. In QG, 48 microbial species changed, with Inmirania increasing by 12 times and Pacificibacter decreasing 19 times. In KZ, 17 microbial species changed, with Jatrophihabitans increasing by 3.7 times and Desulfonatronum decreasing by 11.5 times. In K, 14 microbial species changed, Nitrincola increasing by 4.3 times and Ameyamaea decreasing. In ZJHHL, 55 microbial species changed, with Thermogemmatispora increasing by 21 times and Pacificibacter decreasing by 19 times.

## 4 Discussion

Forest thinning involves the selective removal of poorly growing trees in immature, even-aged stands to enhance the growth conditions for the remaining trees (Lee et al., 2024). This study examined the effects of thinning on physicochemical properties and microbial communities of rhizosphere soil, aiming to optimize thinning practices to improve the growth of trees.

The analysis showed that thinning altered the total nitrogen, organic matter, alkali-hydrolyzed nitrogen, and available potassium contents in the rhizosphere soil of some trees species, indicating a relationship between nutrient changes and thinning (Kim et al., 2023). Microorganisms, which drive soil biogeochemical cycling, likely contribute to these nutrient changes. Notably, thinning significantly affected pH of rhizosphere soil, indicating that alterations in alkaline or acidic substance contents, warranting further investigation into microbial roles in these changes (Zhang et al., 2019).

Further analysis of microbial diversity, richness and community structure, such as SM, MBLRC, BYSF, and etc., showed species-specific responses thinning. This suggests that need to classify potential thinning effects by tree types, requiring larger sample sizes and longer-term monitoring to identify patterns. PCA and PCoA suggested that thinning had no significant effect on the overall microbial community structure, implying stability in soil microecology, though specific bacteria might be related to altered tree growth states due to thinning (Zhang et al., 2023).

Thinning minimally affected the overall composition of rhizosphere soil microbiota, with major microbial species remaining unchanged. Effects were mainly seen in specific microorganisms rather than the primary microbial community. Further analysis showed that the rhizosphere soil microorganisms of shrubs were greatly affected by thinning. Firmicutes in shrub rhizosphere soil was sensitive to thinning, with increased abundance. Firmicutes, include Bacteroides, Bacillus and Pseudomonas, and these have various metabolic pathways and physiological characteristics, and can use a variety of organic and inorganic substances as carbon sources and energy to participate in soil nutrient cycling and ecological function maintenance (Wang et al., 2021; Liu et al., 2024). In addition, rhizosphere soil microbial changes varied by shrub species, necessitating further studies on tree-specific bacterial effects on shrub growth.

At the genus level, thinning effects on rhizosphere soil microorganisms of different tree species were significantly different, highlighting species-specific responses. Shrubs showed higher microbial diversity post-thinning compared to trees, consistent with phylum-level results. We propose that the unique effects of selective logging on rhizosphere soil microorganisms are closely linked to the specific growth traits of different tree species. Each tree species exudes distinct rhizosphere secretions and demonstrates varying capacities for water and nutrient uptake. These differences result in distinct moisture and nutrient profiles within the rhizosphere soil, fostering unique microbial communities for each species (Qiang et al., 2023; Zhang et al., 2023). However, further research is necessary to elucidate the precise mechanisms driving these interactions. Specifically, studies should focus on identifying the specific compounds in rhizosphere exudates that influence microbial composition and understanding how changes in soil moisture and nutrient availability directly affect microbial community dynamics. Unfortunately, our study did not find a consistent pattern in the effects of 10-15% thinning on rhizosphere soil microorganisms, likely due to low precision. Future research using *16S* full-length or metagenomic methods will aim to enhance study precision.

## 5 Conclusion

This study utilized *16S rDNA* high-throughput sequencing technology to examine the effects of 0-15% thinning on the rhizosphere soil microecology of five shrub species and five tree species. The findings indicate that thinning significantly alters the richness and diversity of rhizosphere soil microorganisms, resulting in notable changes in microbial community structure. These shifts in soil microecology may contribute to enhanced tree growth. By elucidating the interaction mechanisms between tree species and microorganisms, this research provides new insights for forest management, ecological restoration and climate change adaptation. However, the study has limitations in scope and lacks specific details on the affected microbial groups and their functional roles. Future research should expand to include a broader variety of species and ecological contexts, incorporate detailed methodological descriptions, and consider the temporal dynamics of microbial community changes. This approach will provide a more comprehensive understanding and practical guidelines for sustainable forest management and ecosystem restoration.

## Data Availability

The data presented in the study are deposited in the NGDC repository, accession number CRA020348.
